# Two Cases of Durable and Deep Responses to Immune Checkpoint Inhibition-Refractory Metastatic Melanoma after Addition of Camu Camu Prebiotic

**DOI:** 10.3390/curroncol30090570

**Published:** 2023-08-25

**Authors:** Steph A. Pang, Arielle Elkrief, Mariana Pilon Capella, Wilson H. Miller

**Affiliations:** 1 Lady Davis Institute and Segal Cancer Centre, Jewish General Hospital, Departments of Medicine and Oncology, McGill University, Montréal, QC H3T 1E2, Canada; 2 Hematology-Oncology Division, Department of Medicine, Centre Hospitalier de l’Université de Montréal (CHUM), Montréal, QC H2X 3E4, Canada; 3 Centre de Recherche du Centre Hospitalier de l’Université de Montréal, Montréal, QC H2X 3E4, Canada

**Keywords:** immune checkpoint inhibitors, melanoma, microbiome, prebiotic, camu camu, rechallenge

## Abstract

Camu camu (CC) is a prebiotic that selectively stimulates growth and activity of beneficial gut microbiota. Work in murine models demonstrated that castalagin, the active compound in CC, preferentially binds to beneficial gut microbiome bacteria, promoting a stronger CD8+T cell anti-cancer response. We present two patients with metastatic melanoma whose cancer progressed on immune checkpoint inhibitors (ICIs) and developed clinically significant immune-related adverse events (irAEs). They were rechallenged with ICIs in combination with CC. The first patient is a 71-year-old woman with metastatic melanoma, whose ICI treatment was complicated by immune-related pneumonitis and colitis. Upon progression on maintenance nivolumab, CC was added to nivolumab, leading to a near complete response (CR). The second patient is a 90-year-old man with recurrent unresectable melanoma, treated with nivolumab, complicated by immune-related rash and diabetes. He developed new subcutaneous calf lesions and a metastatic popliteal lymph node. CC was added to nivolumab. One month later, the patient experienced a CR. Both patients have been on nivolumab and CC with durable responses for more than a year, with minimal irAEs. These two cases suggest that CC may modulate the microbiome, synergizing with ICIs to produce deep, durable responses with minimal irAEs.

## 1. Introduction

Strategies to manipulate the gut microbiota including fecal microbiota transplant and probiotics have shown promise in phase I trials to enhance T cell responses to checkpoint inhibition in solid tumours [1,2,3,4,5]. Another potential microbiome modulator is camu camu (CC), a prebiotic that selectively stimulates growth and activity of beneficial gut microbiota. Evidence from murine models demonstrated that castalagin, the active compound within CC, preferentially binds to beneficial gut bacteria (*Ruminococcus bromii*) and increases their abundance. This promotes an increase in the cluster of differentiation 8 (CD8+)/cluster of differentiation 4 (CD4+)/forkhead box P3 (FOXP3+) T cell ratio and enhances the anti-cancer response to anti-programmed cell death protein 1 (anti-PD-1) agents in solid tumours in mice [6]. A phase I trial is in progress to evaluate CC in combination with immune checkpoint inhibitors (ICIs) in patients with melanoma and non-small cell lung cancer (NCT05303493). Here, we present two patients with metastatic melanoma who had cancer progression on ICIs and who were successfully rechallenged with ICIs and the addition of CC, with minimal immune-related adverse events (irAEs), followed by durable and deep responses.

## 2. Case Description: Patient #1 

This is a 71-year-old woman known for hypertension and osteoarthritis. She presented in 2014 with a right forearm melanoma, B-Raf Proto-Oncogene (BRAF) wildtype, and rat sarcoma virus guanosine triphosphate (RAS) wildtype. She underwent a wide local excision and axillary sentinel lymph node dissection, which was negative for residual malignancy. In 2015, she had a metastatic recurrence to hilar lymph nodes, confirmed by biopsy. 

She was treated on a clinical trial with first line trametinib and durvalumab between 2015 and 2016. After completing 1 year of treatment on protocol, she had an ongoing partial response, and she was continued on active surveillance with no active systemic therapy. Several months later, her disease progressed, with an increasing perihilar mass and significant hemoptysis. She was then retreated on trial with trametinib and durvalumab, and she had another partial response. However, she then developed worsening shortness of breath with lung consolidations on computed tomography (CT) of the chest. During this time, study treatments were held. She underwent a lung biopsy, which demonstrated interstitial pneumonitis and organizing pneumonia. She received moxifloxacin for suspected pneumonia, with no improvement in her dyspnea. Immune-related pneumonitis secondary to ICIs was suspected. The patient was treated with prednisone 1 mg/kg, with resolution of the pneumonitis. She was then tapered off the prednisone over a 1 month period. Subsequently, there was further progression in the size of her perihilar mass to 7.4 × 6.7 cm. Her treatment was then switched to second line nivolumab and recombinant interleukin-2 on trial. She had a best response of stable disease. She was treated with this regimen for 7 months, during which she had no dyspnea or hemoptysis. In November 2018, a CT scan showed progression of disease with a new metastatic adnexal mass. Given its proximity to bowel, the adnexal mass was considered too high a risk to be biopsied, and it was thought to be most likely metastatic melanoma. She received external beam radiation therapy to the adnexal mass. However, after radiation, the adnexal mass continued to grow. In December 2018, she began treatment with third line ipilimumab monotherapy for four cycles, after which she experienced a mixed response. She then underwent debulking surgery and brachytherapy, without complications. She was continued on active surveillance between 2019 and 2020. In 2020, she had recurrent hemoptysis suggestive of recurrent progression, which was confirmed by an increasing perihilar mass on CT scans. She was started on fourth line ipilimumab and nivolumab, with a partial response. After receiving 3 cycles of ipilimumab and nivolumab, she experienced a grade 2 immune-related colitis, manifested as profuse non-bloody diarrhea with recurrent hypomagnesemia. The colitis responded well to prednisone 0.5 mg/kg. Colonoscopy was not performed. Maintenance nivolumab was continued.

**Figure 1:**
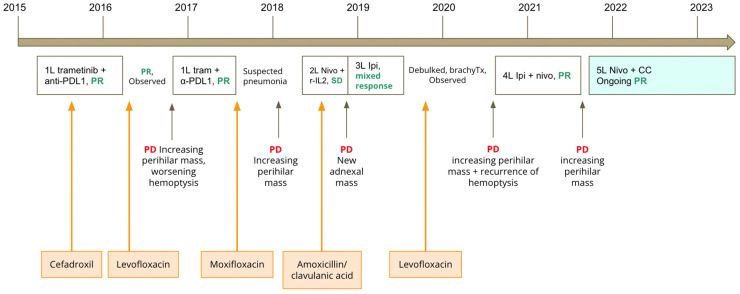
Timeline of immunotherapy treatments, antibiotic use, and clinical evolution of patient 1. Legend: anti-PDL1: anti-programmed death-ligand 1; PR: partial response; PD: progressive disease; tram: trametinib; r-IL2: recombinant interleukin-2; SD: stable disease; Ipi: ipilimumab; brachyTx: brachytherapy; nivo: nivolumab; PR: partial response; CC: camu camu. In orange boxes: antibiotics administered to patient during and in between her treatments. In white boxes: immunotherapy administered to patient, with best clinical response. In green box: treatment with immunotherapy and CC.

**Figure 2:**
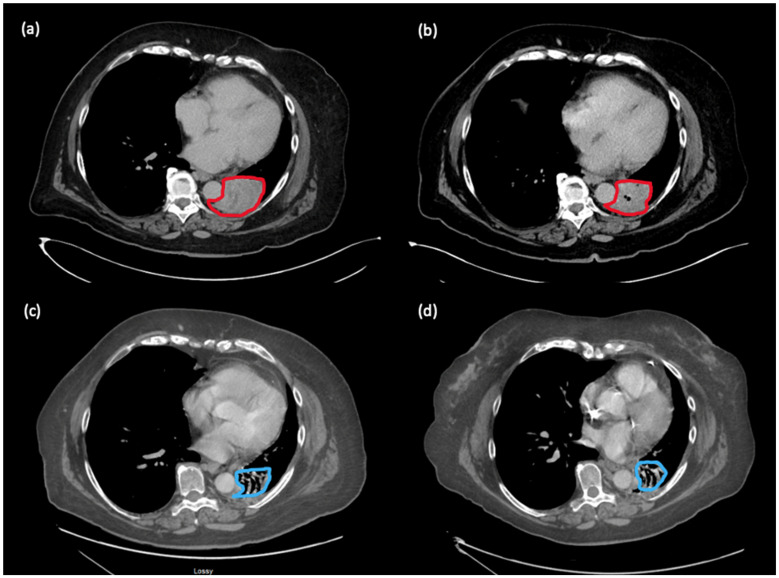
Evolution of perihilar metastasis in patient 1. Outlined in red is tumour; outlined in blue is residual atelectasis/fibrosis as interpreted by radiology. (**a**) July 2021: progression of perihilar mass after 10 cycles of maintenance nivolumab (prior to addition of CC); (**b**) December 2021: improvement in the perihilar mass, shrinking from 6.5 × 4.8 cm to 5.4 × 4.5 cm, after 5 months of treatment with nivolumab and CC; (**c**) November 2022: near complete response on nivolumab and CC; (**d**) February 2023: ongoing near complete response on nivolumab and CC.

In July 2021, after 10 cycles of maintenance nivolumab, there was disease progression in the perihilar mass, increasing from 2.8 × 2.7 cm to 6.5 × 4.8 cm. Treatment options were limited at this stage, and we and the patient had an interest to explore options other than chemotherapy. We considered microbiome modulation in this patient, as she had an extensive antibiotic history throughout her multiple lines of immunotherapy, which may have caused a highly altered gut microbiome. She had received cefadroxil for right calf cellulitis in 2015, levofloxacin in 2016, moxifloxacin in 2017 and amoxicillin/clavulanic acid in 2018 for suspected pneumonias, and levofloxacin in 2019 for bronchitis (see Figure 1 for timing of antibiotics in relation to immunotherapy). Work from our group and other institutions demonstrated that antibiotic use can lead to changes in the gut microbiome associated with worse survival outcomes in patients treated with immunotherapy [7,8,9]. Additionally, our basic science collaborators have used new murine models to show that CC, through its active component castalagin, can modulate the gut microbiota to re-sensitize tumours to ICIs in ICI-refractory mice with solid tumours [6]. Thus, we proposed to the patient to continue nivolumab monotherapy with the addition of 1000 mg of CC ingested orally once daily. This dose was chosen based on the Health Canada-recommended safe dosing range for CC when taken as a natural health supplement, which is 500–1500 mg per day [10]. The patient provided informed consent and proceeded with nivolumab rechallenge with CC. Subsequently, the hemoptysis improved. Scans 5 months later showed improvement in the perihilar mass, shrinking from 6.5 × 4.8 cm to 5.4 × 4.5 cm. Scans conducted in November 2022 demonstrated a near complete response (disappearance of perihilar mass, two 5 mm lung nodules). Scans in February 2023 demonstrated the same ongoing deep response with 18 months of treatment on this regimen (see Figure 2). Clinically, the patient remains asymptomatic from melanoma. She has not had any hemoptysis, which was associated with previous progression of her cancer. Since starting CC, she has not had other irAEs aside from grade 1 non-bloody diarrhea, which has responded well to loperamide.

## 3. Case Description: Patient #2

This is a 90-year-old man known for hypertension, atrial flutter with a permanent pacemaker, parathyroid adenoma, and recurrent resected early-stage squamous cell carcinomas of the skin. The patient was diagnosed with stage II melanoma of the left leg in 2014, which was resected. He underwent multiple resections for recurrent in-transit lesions between 2014 and 2017. In 2017, he had an extensive recurrence in the left calf, which was non-resectable. He then started treatment on a clinical trial with nivolumab combined with an indoleamine 2,3-dioxygenase (IDO) inhibitor. This was complicated by new immune-related diabetes requiring insulin injections and grade 2 immune-related rashes on his trunk, arms, and legs, which responded to topical cortisone creams. He experienced a complete response after 10 cycles of therapy. He was then observed off therapy with resolution of the immune-related rash. 

He had two local recurrences, which were both resected between 2020 and 2021. In February 2021, he had a metastatic recurrence seen on positron emission tomography (PET) scan, with five new foci distal to the left knee. The recurrence was deemed not resectable, and so the patient was then restarted on nivolumab, with no significant local response. In April 2021, a wide local excision of a new lesion above the left knee confirmed metastatic melanoma. A slow local progression of the remaining left calf lesions was seen in June 2021 after 5 cycles, with new subcutaneous hyperpigmented calf lesions and a new fluorodeoxyglucose-avid (FDG-avid) popliteal lymph node on PET scan. After discussing with the patient and obtaining informed consent, a decision was made to continue treatment with nivolumab beyond progression, with the addition of CC at 1500 mg orally once daily. 

**Figure 3:**
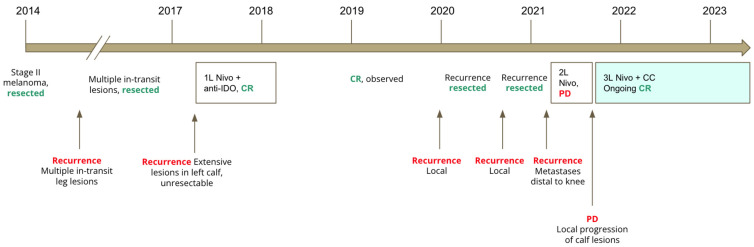
Timeline of immunotherapy treatments and clinical evolution of patient 2. Legend: anti-IDO: indoleamine 2,3-dioxygenase inhibitor; CR: complete response; Nivo: nivolumab; PD: progressive disease; CC: camu camu. In white boxes: immunotherapy administered to patient, with best clinical response. In green box: treatment with immunotherapy and CC.

A PET scan conducted 1 month later demonstrated complete response, with no locoregional/lymph node or distant recurrence. This correlated with the disappearance of calf lesions seen on physical exam. The latest PET scan in April 2023 demonstrated ongoing complete response after 21 months of nivolumab and CC, and the patient has not developed new skin lesions. Additionally, the patient has not had any new irAEs since starting CC. A graphical representation of his clinical evolution is shown in Figure 3. 

## 4. Discussion

Gut microbiome modulation in humans has shown early promising results to enhance T cell responses to checkpoint inhibition in advanced solid tumours, both in the first line setting and the ICI-refractory setting. FMT combined with first line ICIs was studied in the MIMic trial (NCT03772899) [4]. In this Canadian multicentre phase I trial, 20 patients with newly diagnosed advanced melanoma were treated with ICIs in combination with FMT. The primary endpoint of safety was met, with 0% of patients experiencing grade 3 events from FMT alone, and 25% of patients experiencing grade 3 adverse events from combination therapy. A high objective response rate was found, representing 65% of patients (13 out of 20), including 20% of patients (4 out of 20) who experienced complete responses. Correlative studies demonstrated that durable responses were associated with (1) successful long-term strain engraftment in patients from their respective FMT donors; and (2) enriched immunogenic bacteria and loss of deleterious bacteria after FMT. Promising results were also seen in two published phase I trials (NCT03341143, NCT03353402) evaluating FMT with ICIs administered to ICI-refractory patients [1,5]. These trials showed that this strategy was safe and tolerable, and they had objective response rates of 20% and 30%, respectively. 

Other microbiome modulation strategies that have been studied include probiotics and multi-strain microbiome therapeutics. A phase I trial evaluated CBM588, a bifidogenic live bacterial product, in combination with first line ipilimumab and nivolumab in renal cell carcinoma (RCC) (NCT03829111) [2]. In this trial, 30 treatment-naïve patients with metastatic RCC were randomized to receive ipilimumab and nivolumab alone or in combination with the CBM588 probiotic. While the primary endpoint was not met (change in relative abundance of *Bifidobacterium* species), efficacy data suggested that patients receiving CBM588 had higher response rates (58% vs. 20%, *p* = 0.06), and no significant toxicity was observed with the addition of the probiotic. A randomized phase II trial has shown similar results, investigating CBM588 in combination with cabozantinib and ICIs in RCC (NCT05122546) [11]. Patients receiving cabozantinib, nivolumab, and CBM588 had a response rate of 63% compared to 33% in patients receiving cabozantinib and nivolumab alone. Median progression-free survival (PFS) was not reached compared to 5.8 months, respectively (*p* = 0.03). However, the results have been questioned due to the markedly lower median PFS in the cabozantinib/nivolumab compared to historical data [12]. The MET4-IO trial (NCT03686202) is an early phase trial evaluating a cultivated, orally delivered 30-species microbial consortium (Microbial Ecosystem Therapeutic 4, MET4) isolated from the stool of a healthy donor [13]. MET4 was delivered to patients with advanced solid tumours in combination with ICIs, and the trial achieved its primary safety and tolerability outcomes. While the primary ecological endpoints (MET4 relative abundance, change from baseline, number of taxa >1%, Shannon diversity, and observed operational taxonomic units) were not met, increased relative abundance of several MET4 bacteria, such as *Enterococcus* and *Bifidobacterium*, were observed; in other previous studies, these bacteria were found to be associated with ICI responsiveness [13,14,15]. 

The results of our patients in this case series support a potential novel, easily accessible strategy of microbiome modulation for immunotherapy-refractory patients, which has been confirmed in prospective studies (NCT05303493). We presented two cases of patients with metastatic melanoma who had disease progression on ICIs and then durable deep responses with the continuation of ICIs and the addition of CC. These cases support preclinical models of CC or its active component, castalagin, enabling re-sensitization to ICIs. Both patients had previous responses to ICIs (partial response and complete response as best responses), and it is unlikely that the tumour response with addition of CC was due to pseudoprogression, given that both patients were treated with ICIs for at least 5 months prior to progression. It is possible that the gut microbiome had been altered unfavourably in these patients, thus leading to progression on ICIs. For example, during her previous lines of therapy, patient #1 received five courses of antibiotics (cefadroxil, levofloxacin, moxifloxacin, amoxicillin–clavulanate) for cellulitis and respiratory infections, which may have caused significant alterations in her gut microbiome. This is consistent with extensive published data demonstrating an association of antibiotic use with ICIs and worse survival outcomes, particularly in melanoma and renal cell carcinoma, which was confirmed in systematic reviews and meta-analyses [7,8,9].

In mouse models, CC supplementation is associated with antitumour activity that is microbiome-dependent and circumvents the anti-PD-1 resistance found in mice that received fecal matter transplant (FMT) from non-responsive patients with non-small cell lung cancer (NSCLC) [6]. Castalagin polyphenol was identified as the active molecule, slowing tumour growth in mice that received FMT from non-responsive patients. It was found to shift microbiota composition to favour *Ruminococcus bromii*, which is associated with improved anti-tumour responses, by preferentially binding to the cellular membrane of *Ruminococcus bromii* and increasing its abundance. Abundance of this bacteria has been correlated with the dose-dependent anti-tumour effect of castalagin [6]. 

This case report supports further study of CC as a less expensive, less cumbersome, and more easily scalable alternative to FMT for immunomodulation of the microbiome to achieve improved responses to ICIs. It may also have a lower risk of adverse effects, compared to FMT, which is known to be associated with diarrhea, risk of transmitted infections, and hypophosphatemia [3,4]. Current published data on adverse effects of CC are limited. However, one published human study comprising 20 healthy volunteers taking CC supplementation demonstrated no adverse biological or metabolic events while exerting antioxidative and anti-inflammatory effects on subjects [16]. Per Health Canada’s Licensed Natural Health Product Database, various minor gastrointestinal effects may occur at the recommended dose of 500–1500 mg per day, including diarrhea, nausea, and abdominal cramps, due to the osmotic effect of unabsorbed Vitamin C in the gastrointestinal tract [10]. 

Of note, the rapidity of progressive disease may be a limiting factor for successful use of prebiotic modulation of the microbiome for patients with ICI-refractory cancer. Both of our patients had progression, but not rapidly progressive disease. Therefore, we were able to continue with immunotherapy while waiting for the effects of re-sensitization to ICIs by microbiome modulation.

Given that our two patients had histories of significant irAEs prior to ICIs, their minimal irAEs on ICIs with CC raise interesting questions about irAEs and microbiome modulation with CC. In vitro and in vivo models show a correlation of CC with downregulated expression of proinflammatory cytokines and chemokines, such as those associated with the mitogen-activated protein kinases/activator protein-1 pathway, the nuclear factor kappa-light-chain-enhancer of activated B cells pathway, and the nuclear factor of activated T cells pathway [16,17]. Decreased systemic inflammation associated with CC could thus potentially contribute to lower rates of irAEs. Further research is needed to better understand the relationship between CC- and castalagin-mediated microbiome modulation and its impact on T cell activity.

## 5. Conclusions

CC is a prebiotic that has demonstrated an additive effect to anti-PD-1 activity in mouse tumour models. There is evidence that the active component of CC, castalagin, shifts the microbiota composition and the systemic metabolite profile, and it has the potential to circumvent anti-PD-1 resistance in mouse models. Here, we presented two patients with ICI-refractory metastatic melanoma, who experienced durable CR/near CR after CC was added to their ICI regimen, with minimal irAEs. A phase I clinical trial is underway to further evaluate the therapeutic role of CC for ICI-refractory advanced melanoma and CC added to standard-of-care first line ICIs for advanced melanoma and NSCLC (NCT05303493). CC, or the active ingredient, castalagin, may prove to be a cost-effective, easily administered microbiome-modulating agent that synergizes with ICIs to induce and maintain durable tumour responses to ICIs. 

## Data Availability

Data about the patients were obtained from case reviews of their electronic health records in Montréal, Canada. Copies of the consent forms signed by the two patients are available upon request from the authors.
